# Assessing Large Language Models for Medical Question Answering in Portuguese: Open-Source Versus Closed-Source Approaches

**DOI:** 10.7759/cureus.84165

**Published:** 2025-05-15

**Authors:** João Abrantes

**Affiliations:** 1 Imaging Department, Unidade Local de Saúde de Trás-os-Montes e Alto Douro, Vila Real, PRT; 2 Data Science and Artificial Intelligence, University of London, London, GBR

**Keywords:** artificial intelligence in medicine, large language models, medical question answering, portuguese language, prompt engineering

## Abstract

Large language models (LLMs) show promise in medical knowledge assessment. This study benchmarked a closed-source (GPT-4o, OpenAI, San Francisco, CA) and an open-source (LLaMA 3.1 405B, Meta AI, Menlo Park, CA) LLM on 148 multiple-choice questions from the 2023 Portuguese National Residency Access Examination across five clinical domains. Using five distinct prompting strategies, models provided single-best-answer predictions. GPT-4o consistently outperformed LLaMA 3.1 by 7-11% accuracy across all prompts. Chain-of-thought prompting yielded the highest numerical accuracy for GPT-4o, though this improvement was not statistically significant over simpler prompts in post-hoc analyses, while offering minimal benefit when applied to LLaMA 3.1. Both models performed best in pediatrics and less accurately in surgery and psychiatry questions. Bias assessment indicated GPT-4o aligned well with correct answer distributions, unlike LLaMA 3.1, which showed prompt-dependent skew. Closed-source models currently demonstrate higher accuracy on Portuguese medical questions, likely due to extensive training. However, open-source models remain valuable for data control, though domain-focused fine-tuning may be needed for optimal performance in high-stakes applications.

## Introduction

Large language models (LLMs) represent a significant advancement in artificial intelligence (AI), capable of understanding input prompts and generating human-like text across various complex tasks [1,2]. Their proficiency in processing and generating vast amounts of textual data makes them particularly promising for application within the medical field [3]. The interaction with these models relies heavily on "prompting," the act of providing instructions to guide their output, making prompt engineering crucial for optimizing performance [4].

A primary application and evaluation method for LLMs in medicine involves assessing their performance on standardized medical examinations, such as licensing or board exams [5]. Recent studies have shown that models like GPT-3.5 and particularly GPT-4 can achieve high accuracy, sometimes meeting or exceeding passing thresholds on challenging exams like the United States Medical Licensing Examination (USMLE) [6-8]. Similar capabilities have been observed in non-English contexts, such as GPT-4 achieving high scores on the Brazilian National Examination for Medical Degree Revalidation [9]. However, the overall performance landscape can show variability, and results are not always consistent across different models or datasets [10].

Despite these advancements, several gaps remain. The majority of LLM research and development has centered on the English language, leaving performance in other languages, like Portuguese, relatively underexplored [10]. Performance can vary widely depending on language resource levels and representation in training data [11,12]. Healthcare institutions face a choice between using proprietary, closed-source models (often offering state-of-the-art performance but lacking transparency and requiring data transfer) and open-source models (allowing for greater data control, privacy, customization, and transparency, but potentially lagging in performance) [13,14]. Direct comparisons in specific clinical contexts are needed to inform deployment decisions. Additionally, while prompt engineering techniques, such as chain-of-thought (CoT) prompting, which encourages step-by-step reasoning [15], can influence LLM outputs [16,17], their effectiveness varies significantly depending on the model, task complexity, and specific domain, including medicine [18].

This study aimed to address the identified gaps by benchmarking and comparing the performance of a leading closed-source LLM (GPT-4o, OpenAI, San Francisco, CA) against a large, state-of-the-art open-source LLM (LLaMA 3.1 405B, Meta AI, Menlo Park, CA). The evaluation was conducted using multiple-choice questions from the 2023 Portuguese National Residency Access Examination (PNA), a high-stakes standardized test for medical graduates in Portugal [19,20]. Our primary objectives were to (1) compare the overall accuracy of GPT-4o and LLaMA 3.1 on these Portuguese medical questions, (2) assess the impact of five distinct prompting strategies on each model's performance, and (3) evaluate performance variations across five key medical domains.

## Materials and methods

Dataset and preprocessing

The evaluation benchmark utilized was derived from the 2023 Prova Nacional de Acesso, a nationwide Portuguese multiple-choice examination used to rank medical graduates for specialty training positions [19]. The official 2023 PNA exam (150 questions, five single-best-answer options, Portuguese language) was obtained from public ACSS (Administração Central do Sistema de Saúde) sources. A Python script parsed the source PDF, extracting question text, options, and answer keys. Two questions requiring image interpretation were excluded, yielding a final dataset of 148 text-only questions. Data integrity was verified against the source PDF.

Questions were assigned to one of five medical domains (medicine, surgery, pediatrics, gynecology/obstetrics, and psychiatry) using a semi-automated process: initial categorization by the study's LLMs was reviewed and finalized by a domain expert where necessary. The final dataset was structured as a CSV file.

Large language models used

Two LLMs were selected based on their high ranking for Portuguese on the Open Portuguese LLM leaderboard (January 2025) [21]: OpenAI’s GPT-4o (closed-source) and Meta AI’s LLaMA 3.1 405b instruct (open-source). For GPT-4o, the specific version queried via OpenRouter was openai/gpt-4o, and for LLaMA 3.1, the version was meta-llama/llama-3.1-405b-instruct (latest available versions during the experimental period, January 2025). Both were accessed via the OpenRouter API (application programming interface) and used "out-of-the-box" without study-specific fine-tuning, and all API calls were made with default settings for temperature.

Prompting strategies

All instructional prompts were provided in English, while the exam questions were in Portuguese. This approach was chosen to maintain consistency in the instructional cues given to the models, which are predominantly trained on English data. Five prompt styles were tested in English: raw (question and options only); brief instruction (BI; short task instruction prepended); long instruction (LI; detailed task description prepended); chain-of-thought (CoT; prepended instruction to "think step by step"); and question-specific automatic prompt generation (QAPG; model generates its own prompt first) [4]. All prompts required the output format "Final Answer: X" (where X is A-E).

Experimental procedure

A Python script systematically submitted each of the 148 questions to both LLMs using all five prompt styles. To account for response variability, this process was repeated five times per condition, totaling 7,400 evaluations. Model outputs (answer choice A-E) were logged, and correctness was determined against the ground truth answer key. The evaluation was performed in a zero-shot knowledge setting.

Outcome measures and statistical analysis

The primary outcome was accuracy (proportion of correct answers). Statistical comparisons were performed as follows: GPT-4o vs. LLaMA 3.1 performance using the Wilcoxon signed-rank test; prompt style effects within each model using the Friedman test with Nemenyi post-hoc tests; accuracy differences across medical domains using the Kruskal-Wallis test with Mann-Whitney U post-hoc tests; and answer choice bias using Spearman correlation between predicted and actual correct answer distributions. A significance level of α = 0.05 was used for all tests. Analyses were conducted using Python version 3.12 (Python Software Foundation, Wilmington, Delaware).

## Results

Overall model performance by prompting strategy

Accuracy results for both models across the five prompting strategies are stated in Table 1. GPT-4o consistently achieved higher accuracy than LLaMA 3.1 405b instruct across all tested prompt styles, with accuracy margins ranging from approximately 7% to 11%. This difference in performance was statistically significant (Wilcoxon signed-rank test, p < 0.0001).

**Table 1:** Comparison of mean accuracy between GPT-4o and LLaMA 3.1 405b instruct across five prompting styles. Accuracy (%) represents the mean percentage of correct answers across 148 questions and five experimental iterations for each condition. Models compared are OpenAI's GPT-4o and Meta AI's LLaMA 3.1 405b instruct. Prompt styles tested include: raw, brief instruction, long instruction, chain-of-thought, and question-specific automatic prompt generation (QAPG). The final column shows the p-value from the Wilcoxon signed-rank test comparing the accuracy distributions of GPT-4o and LLaMA 3.1 for each specific prompt style (α = 0.05).

Prompt style	GPT-4o accuracy (%)	LLaMA accuracy (%)	Wilcoxon (GPT-4o vs. LLaMA) p-value
Raw	86.49	78.38	0.0186
Brief instruction	88.51	79.73	0.0093
Long instruction	87.16	79.05	0.0233
Chain-of-thought	89.19	78.38	0.0017
QAPG	83.11	71.62	0.0031

Within-model performance varied significantly across prompt styles for both GPT-4o (Friedman test, χ2 = 16.97, p = 0.0020) and LLaMA 3.1 (Friedman test, χ2 = 13.41, p = 0.0094). For GPT-4o, the CoT prompt yielded the highest numerical accuracy (89.19%), followed by BI (88.51%). The QAPG style resulted in the lowest accuracy for GPT-4o (83.11%). For LLaMA 3.1, prompt engineering yielded minimal gains; BI resulted in the highest accuracy (79.73%), slightly above the raw baseline (78.38%), while CoT showed no improvement over the baseline (78.38%). QAPG significantly degraded LLaMA 3.1 performance, yielding the lowest accuracy (71.62%).

Post-hoc Nemenyi tests revealed no statistically significant pairwise differences between raw, BI, LI, and CoT prompts for GPT-4o (Table 2) or LLaMA 3.1 (Table 3).

**Table 2:** Pairwise Nemenyi post-hoc test results for accuracy differences across prompt styles for GPT-4o. The table shows adjusted p-values from the Nemenyi post-hoc test for pairwise comparisons of accuracy distributions across the five prompt styles for OpenAI's GPT-4o model. Prompt styles tested include: raw, brief instruction, long instruction, chain-of-thought, and question-specific automatic prompt generation (QAPG). No pairwise comparisons reached statistical significance (α = 0.05).

	Raw (baseline)	Brief instruction	Long instruction	Chain-of-thought	QAPG
Raw (baseline)	1.000000	0.998736	0.999984	0.996114	0.990840
Brief instruction	0.998736	1.000000	0.999745	0.999984	0.948303
Long instruction	0.999984	0.999745	1.000000	0.998736	0.981797
Chain-of-thought	0.996114	0.999984	0.998736	1.000000	0.922325
QAPG	0.990840	0.948303	0.981797	0.922325	1.000000

**Table 3:** Pairwise Nemenyi post-hoc test results for accuracy differences across prompt styles for LLaMA 3.1 405b instruct. The table shows adjusted p-values from the Nemenyi post-hoc test for pairwise comparisons of accuracy distributions across the five prompt styles for Meta AI's LLaMA 3.1 405b instruct model. Prompt styles tested include: raw, brief instruction, long instruction, chain-of-thought, and question-specific automatic prompt generation (QAPG). No pairwise comparisons reached statistical significance (α = 0.05).

	Raw (baseline)	Brief instruction	Long instruction	Chain-of-thought	QAPG
Raw (baseline)	1.000000	0.999745	0.999984	1.000000	0.889692
Brief instruction	0.999745	1.000000	0.999984	0.999745	0.805149
Long instruction	0.999984	0.999984	1.000000	0.999984	0.850483
Chain-of-thought	1.000000	0.999745	0.999984	1.000000	0.889692
QAPG	0.889692	0.805149	0.850483	0.889692	1.000000

Performance across medical domains

**Figure 1:**
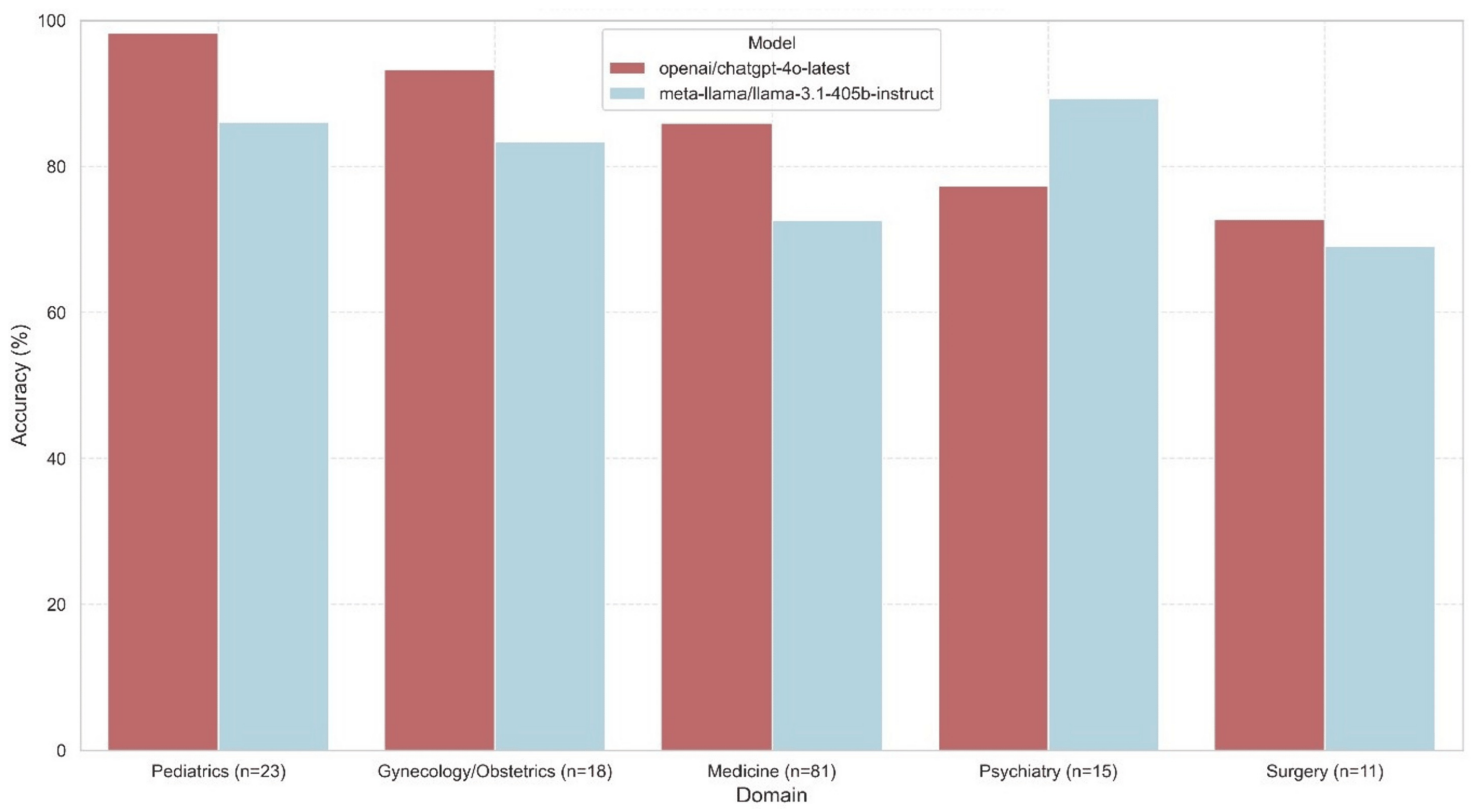
Accuracy by medical domain and model. Mean accuracy (%) of OpenAI's GPT-4o and Meta AI's LLaMA 3.1 405b instruct across five medical domains derived from the 2023 Portuguese National Residency Access Examination.

Model performance varied significantly across the five medical domains for both GPT-4o (Kruskal-Wallis test, H = 32.32, p < 0.0001) and LLaMA 3.1 (Kruskal-Wallis test, H = 20.39, p = 0.0004). Overall domain performance is visualized in Figure 1 and stated in Table 4.

**Table 4:** Detailed accuracy by medical domain, model, and prompt style. The table shows detailed mean accuracy (%) for OpenAI's GPT-4o and Meta AI's LLaMA 3.1 405b instruct across five medical domains and five prompt styles. Accuracy represents the mean percentage of correct answers across five experimental iterations for each condition within each domain. QAPG: question-specific automatic prompt generation.

Final domain	Model	Prompt style	Accuracy (%)
Gynecology/obstetrics	LLaMA 3.1 405b	Raw	83.33
		Brief instruction	83.33
		Long instruction	83.33
		Chain-of-thought	83.33
		QAPG	83.33
	GPT-4o	Raw	94.44
		Brief instruction	94.44
		Long instruction	94.44
		Chain-of-thought	94.44
		QAPG	88.89
Medicine	LLaMA 3.1 405b	Raw	71.6
		Brief instruction	74.07
		Long instruction	75.31
		Chain-of-thought	74.07
		QAPG	67.9
	GPT-4o	Raw	85.19
		Brief instruction	87.65
		Long instruction	85.19
		Chain-of-thought	87.65
		QAPG	83.95
Pediatrics	LLaMA 3.1 405b	Raw	91.3
		Brief instruction	91.3
		Long instruction	86.96
		Chain-of-thought	82.61
		QAPG	78.26
	GPT-4o	Raw	100.0
		Brief instruction	100.0
		Long instruction	100.0
		Chain-of-thought	100.0
		QAPG	91.3
Psychiatry	LLaMA 3.1 405b	Raw	93.33
		Brief instruction	93.33
		Long instruction	93.33
		Chain-of-thought	93.33
		QAPG	73.33
	GPT-4o	Raw	73.33
		Brief instruction	80.0
		Long instruction	80.0
		Chain-of-thought	80.0
		QAPG	73.33
Surgery	LLaMA 3.1 405b	Raw	72.73
		Brief instruction	72.73
		Long instruction	63.64
		Chain-of-thought	72.73
		QAPG	63.64
	GPT-4o	Raw	72.73
		Brief instruction	72.73
		Long instruction	72.73
		Chain-of-thought	81.82
		QAPG	63.64

GPT-4o achieved its highest accuracy in pediatrics (mean of 100.00% across most prompts) and gynecology/obstetrics (mean of 94.44% across most prompts). Its lowest performance was observed in surgery (mean accuracy ranging from 63.64% with QAPG to 81.82% with CoT) and psychiatry (mean accuracy ranging from 73.33% with raw/QAPG to 80.00% with other prompts). LLaMA 3.1 also performed best in pediatrics (mean accuracy up to 91.30%) and showed relatively strong performance in psychiatry (mean of 93.33% across most prompts, dropping with QAPG). Its weakest performance was in medicine (accuracy ranging from 67.90% to 75.31%) and surgery (accuracy ranging from 63.64% to 72.73%).

Post-hoc Mann-Whitney U tests revealed significant differences between specific domain pairs for both models (Table 5), notably LLaMA 3.1's significantly lower accuracy in medicine compared to pediatrics, psychiatry, and gynecology/obstetrics. GPT-4o showed significant differences between domains, such as pediatrics vs. surgery/psychiatry and gynecology/obstetrics vs. surgery/psychiatry.

**Table 5:** Pairwise Mann-Whitney U test results for accuracy differences between medical domains. The table shows p-values from post-hoc pairwise Mann-Whitney U tests comparing accuracy distributions between different medical domains for each model (OpenAI's GPT-4o and Meta AI's LLaMA 3.1 405b instruct). * indicates p < 0.05, representing a statistically significant difference in accuracy between the compared domains for that model (α = 0.05).

Comparison	LLaMA 3.1	GPT-4o
Gynecology/obstetrics vs. medicine	0.0346*	0.0568
Gynecology/obstetrics vs. pediatrics	0.5872	0.0719
Gynecology/obstetrics vs. psychiatry	0.2705	0.0033*
Gynecology/obstetrics vs. surgery	0.0458*	0.0007*
Medicine vs. pediatrics	0.0030*	0.0002*
Medicine vs. psychiatry	0.0021*	0.0587
Medicine vs. surgery	0.5875	0.0116*
Pediatrics vs. psychiatry	0.5129	0.0000*
Pediatrics vs. surgery	0.0090*	0.0000*
Psychiatry vs. surgery	0.0040*	0.5506

Answer choice bias assessment

The alignment between the distribution of predicted answer choices (A-E) and the actual distribution of correct answers was assessed using Spearman correlation (Table 6). The distribution of correct answers in the dataset was skewed toward options D (37) and E (34), with fewer instances of A (25), B (26), and C (26).

**Table 6:** Spearman correlation assessing answer choice bias. Spearman correlation coefficients (ρ) and corresponding p-values comparing the frequency distribution of predicted answer choices (A–E) to the frequency distribution of the correct answers in the dataset (n = 5 answer choice categories). Correlations were calculated separately for OpenAI's GPT-4o and Meta AI's LLaMA 3.1 405b instruct under each of the five prompt styles. * p < 0.05 statistically significant correlations (α = 0.05). QAPG: question-specific automatic prompt generation.

Prompt style	GPT-4o ρ	GPT-4o p-value	LLaMA 3.1 ρ	LLaMA 3.1 p-value
Raw	1.0	<0.0001*	0.9	0.0374*
Brief instruction	0.9	0.0374*	0.9	0.0374*
Long instruction	0.9	0.0374*	0.8721	0.0539
Chain-of-thought	0.9	0.0374*	0.3591	0.5528
QAPG	0.9	0.0374*	0.3	0.6238

GPT-4o demonstrated a consistently strong and statistically significant positive correlation (ρ ≥ 0.90, p < 0.05) between its predicted answer distribution and the ground truth distribution across all five prompting styles. In contrast, LLaMA 3.1 showed variability depending on the prompt style. While raw and BI prompts yielded strong, significant correlations (ρ = 0.90, p = 0.0374), the correlations became weaker and non-significant for LI (ρ = 0.87, p = 0.0539), CoT (ρ = 0.36, p = 0.5528), and QAPG (ρ = 0.30, p = 0.6238).

## Discussion

This study investigated the comparative performance of a leading closed-source (GPT-4o) and a large open-source (LLaMA 3.1 405b) LLM on Portuguese medical board-style questions, considering the impact of various prompting strategies. Our results indicate that GPT-4o consistently outperformed LLaMA 3.1 across all conditions, suggesting proprietary models currently hold an advantage in this specific task. Prompt engineering yielded model-dependent effects: CoT prompting offered a slight numerical benefit for GPT-4o, yielding the highest mean accuracy, though this was not statistically significant in post-hoc comparisons against simpler prompts, while a complex automatic prompt generation strategy (QAPG) proved detrimental to both, particularly LLaMA 3.1. Performance also varied significantly by medical domain for both models, and LLaMA 3.1 exhibited greater susceptibility to prompt-induced answer choice bias compared to the more stable GPT-4o.

The observed performance gap between GPT-4o and LLaMA 3.1 aligns with findings suggesting that state-of-the-art closed-source models, likely benefiting from vast proprietary training data and sophisticated fine-tuning procedures including reinforcement learning from human feedback, often demonstrate superior capabilities on complex tasks compared to even very large open-source counterparts [7,8]. While LLaMA 3.1 represents a significant advancement in open-source LLMs, our findings suggest that closing the performance gap in specialized, non-English domains like Portuguese medical question answering may require further focused tuning [6]. The varied impact of prompting strategies underscores the sensitivity of LLMs to input phrasing [4,15]. The numerical benefit of CoT for GPT-4o, despite statistical non-significance in pairwise tests, hints at its potential to elicit better reasoning in highly capable models, whereas its lack of impact on LLaMA 3.1 might reflect differences in underlying training or architectural capacity to leverage such reasoning prompts effectively [22]. The poor performance of the QAPG approach suggests that complex, multi-step, or automatically generated prompts can sometimes hinder rather than help, potentially confusing models not specifically tuned for such meta-instructions [4,23].

Domain-related accuracy discrepancies observed here, with both models performing better in areas like pediatrics and less well in surgery or psychiatry, resonate with literature noting challenges for LLMs in certain nuanced medical specialties [8]. This may reflect gaps in knowledge representation acquired during pre-training or differing complexities in clinical reasoning required across domains [11,24]. LLaMA 3.1's particularly sharp dips in medicine and surgery suggest that while large open-source models possess considerable knowledge, robust performance on highly specialized exam content might necessitate specific fine-tuning strategies [25]. The bias assessment findings are also important as GPT-4o's consistent alignment with the ground truth answer distribution across prompts suggests higher reliability in this aspect, whereas LLaMA 3.1's prompt-dependent skew highlights potential risks in deploying models whose output distributions might be easily perturbed by instructional nuances.

This study provides a direct comparison between leading open- and closed-source models in a non-English, high-stakes medical context, extending previous evaluations often focused on English datasets like USMLE [9]. The relatively strong performance of LLaMA 3.1 in certain domains like pediatrics and psychiatry confirms the potential of open-source alternatives, suggesting targeted specialization could yield further improvements [26].

However, the study has limitations, such as the use of a single exam dataset (PNA 2023) from one year, which limits generalizability, as question styles, content focus, and difficulty may vary across years or other question banks [20]. Evaluating models "out-of-the-box" without domain-specific fine-tuning highlights baseline capabilities but likely underestimates their peak potential performance. A significant limitation is the use of English instructional prompts for Portuguese questions. This decision, made to standardize the instructional input based on the primary training language of most LLMs, may have inadvertently favored models like GPT-4o, which possess strong English understanding and cross-lingual capabilities. The potential impact of this language mismatch on the performance of LLaMA 3.1 warrants further investigation, perhaps by using translated prompts in future work. The division of the 148 questions into five clinical domains resulted in small subgroup sizes for some specialties, which may magnify observed differences and limit the robustness of domain-specific conclusions.

The findings have practical implications for healthcare institutions considering LLM adoption. While closed-source models like GPT-4o may offer superior accuracy currently, factors such as data privacy, security, regulatory compliance, cost, and the ability to customize and retain control favor the exploration and potential deployment of open-source alternatives, especially if models can be run locally [13,14,27]. Future research should prioritize domain-specific fine-tuning of large open-source models like LLaMA 3.1 on Portuguese medical corpora to assess if the performance gap can be narrowed. Incorporating larger, more diverse Portuguese medical datasets and exploring adaptive or hybrid prompting techniques are also crucial next steps. Evaluating the clinical plausibility and factual correctness of model outputs by human clinicians would provide valuable insights beyond automated accuracy scores [24].

## Conclusions

This investigation compared the capabilities of leading closed-source and open-source LLMs in the context of Portuguese medical examination questions. The results indicated a clear performance advantage for the closed-source model under the tested conditions. Furthermore, the study demonstrated that the choice of prompting strategy significantly influences model accuracy and output consistency, with the open-source model showing greater variability.

These results highlight that while proprietary models may currently offer higher baseline accuracy for specific medical tasks, careful consideration of prompt design is crucial for all LLMs. For open-source models, achieving reliable performance comparable to closed-source counterparts in specialized domains likely necessitates further development, potentially through targeted fine-tuning on relevant medical corpora, to ensure their suitability for high-stakes applications.
